# Characterization of the complete plastid genome of a Chinese endemic species *Carya kweichowensis*

**DOI:** 10.1080/23802359.2018.1464414

**Published:** 2018-04-23

**Authors:** Linjiang Ye, Chaonan Fu, Yuehua Wang, Jie Liu, Lianming Gao

**Affiliations:** a School of Life Sciences, Yunnan University, Kunming, China;; b Key Laboratory for Plant Diversity and Biogeography of East Asia, Kunming Institute of Botany, Chinese Academy of Sciences, Kunming, China;; c University of Chinese Academy of Sciences, Beijing, China

**Keywords:** *Carya kweichowensis*, conservation genetics, endangered species, genome skimming, plastid genome

## Abstract

*Carya kweichowensis* is an importantly economic tree species in Juglandaceae, which is critically endangered and endemic to Guizhou, China. The plastid genome of *C. kweichowensis* was assembled and characterized based on Illumina pair-end sequencing data using genome skimming approach. The complete plastid genome is 175,313 bp in length, with a GC content of 35.8%. It is a typical quadripartite structure, containing a large single copy (LSC) region of 89,858 bp and a small single copy (SSC) region of 3569 bp separated by a pair of extremely extensive inverted repeats (IRs) of 40,943 bp. A total of 142 genes were annotated, including 94 protein-coding genes, 40 tRNA genes, and eight rRNA genes. A maximum likelihood (ML) phylogenetic tree on plastid genomes revealed that *C. kweichowensis* is close to *Annamocarya sinensis*. The newly characterized complete plastid genome of *C. kweichowensis* will provide essential data for further studies of this endangered species.

The Guizhou hickory, *Carya kweichowensis* Kuang & A. M. Lu ex Chang & Lu, is a deciduous tree species with pinnately compound leaves and small nuts, narrowly scattered in subtropical evergreen broad-leaf forest and endemic to Guizhou, China (Zhang et al. 2013). However, due to its highly economic values of high-quality timber and nutritious nuts, the wild resources of *C. kweichowensis* have been anthropogenically over-exploited, and its natural habitats were fragmented. Recently, *C. kweichowensis* was ranked as critically endangered (CR) of the Red List of China Higher Plants based on IUCN Red List Categories and Criteria (Qin et al. 2017). The native populations of the species have dramatically declined in past decades, which need extremely urgent conservation. Hence it is important to set up appropriate conservation strategies to conserve the genetic diversity of the species.

In this study, we assembled and characterized the complete plastid genome of *C. kweichowensis* via genome skimming approach (Straub et al. 2012). Sequence data have been deposited in GenBank (Accession Number: MH121170).

We sampled a natural population with fresh, healthy leaves of *C. kweichowensis* from Anlong county of Guizhou, China (N 25°02′19″, E 105°14′44″). A single individual was selected for total genomic DNA isolation using CTAB method (Doyle and Doyle 1987). Voucher specimen (Liuj167640) was deposited in the Herbarium of Kunming Institute of Botany (KUN), Chinese Academy of Sciences. Illumina paired-end (PE) library was constructed from fragmented genomic DNA following the standard protocols (NEBNext^®^ Ultra II™DNA Library Prep Kit for Illumina^®^), and sequenced on the Illumina HiSeq X Ten platform (Illumina, San Diego, CA). Approximately, 2.2 Gb of high quality clean data with adaptors trimmed and quality filtered were generated. The plastid genome PE reads were aligned to the reference (GenBank Accession: KX703001) using bowtie2 v2.2.6 (Langmead and Salzberg 2012), and assembled into a genome using SPAdes v3.9.0 (Bankevich et al. 2012). The annotation of the assembled plastid genome was performed with Dual Organellar Genome Annotator (DOGMA) (Wyman et al. 2004).

The complete plastid genome of *C. kweichowensis* was 175,313 bp in length, showing a typical quadripartite structure: a pair of inverted repeats (IRs) of 40,943 bp separating by a large single copy (LSC) region of 89,858 bp and a small single copy (SSC) region of 3569 bp. The GC content of the plastid genome was 35.8%. This plastid genome contained 142 genes, including 94 protein-coding genes, eight ribosomal RNA (rRNA) genes, and 40 transfer RNA (tRNA) genes. The phylogenetic relationship of *C. kweichowensis* was conducted based on the complete plastid genome sequences of 19 species in Fagales (Table S1), and the maximum-likelihood (ML) analysis was conducted using RAxML v 8.0 (Stamatakis 2014). The phylogenetic relationships of the 20 species were well resolved with strongly supported (Figure 1).

**Figure 1.**
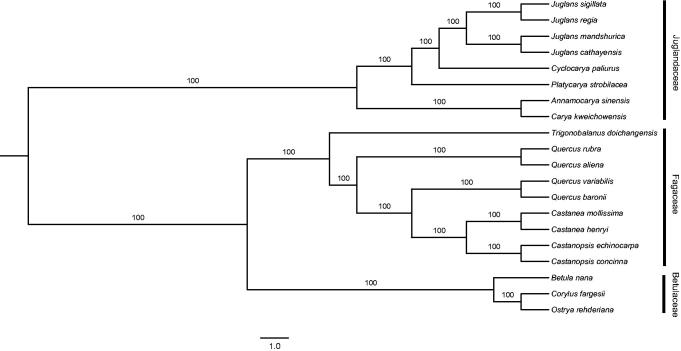
Maximum-likelihood (ML) phylogenetic tree based on complete plastid genome sequences of the 20 species.

The newly characterized complete plastid genome of *C. kweichowensis* will provide essential resources for further study on the evolution and genetic diversity of the endemic species, as well as provide fundamental information to effectively conserve the CR species.

## References

[CIT0001] Bankevich A, Nurk S, Antipov D, Gurevich AA, Dvorkin M, Kulikov AS, Pyshkin AV. 2012 SPAdes: a new genome assembly algorithm and its applications to single-cell sequencing. J Comput Biol. 19:455–477. 22506599 10.1089/cmb.2012.0021 PMC3342519

[CIT0002] Doyle JJ, Doyle JL. 1987 A rapid DNA isolation procedure for small quantities of fresh leaf tissue. Phytochem Bull Bot Soc Am. 19:11–15.

[CIT0003] Langmead B, Salzberg SL. 2012 Fast gapped-read alignment with Bowtie 2. Nat Methods. 9:357–359. 22388286 10.1038/nmeth.1923 PMC3322381

[CIT0004] Qin HN, Yang Y, Dong SY, He Q, Jia Y, Zhao LN, Yu SX, Liu HY, Liu B, Yan YH, et al 2017 Threatened species list of China’s higher plants. Biodivers Sci. 25:696–744.

[CIT0005] Stamatakis A. 2014 RAxML version 8: a tool for phylogenetic analysis and post-analysis of large phylogenies. Bioinformatics. 30:1312–1313. 24451623 10.1093/bioinformatics/btu033 PMC3998144

[CIT0006] Straub SC, Parks M, Weitemier K, Fishbein M, Cronn RC, Liston A. 2012 Navigating the tip of the genomic iceberg: next-generation sequencing for plant systematics. Am J Bot. 99:349–364. 22174336 10.3732/ajb.1100335

[CIT0007] Wyman SK, Jansen RK, Boore JL. 2004 Automatic annotation of organellar genomes with DOGMA. Bioinformatics. 20:3252–3255. 15180927 10.1093/bioinformatics/bth352

[CIT0008] Zhang JB, Li RQ, Xiang XG, Manchester SR, Lin L, Wang W, Wen J, Chen ZD. 2013 Integrated fossil and molecular data reveal the biogeographic diversification of the eastern Asian-eastern North American disjunct hickory genus (*Carya* Nutt.). PLoS ONE. 8:e70449. 23875028 10.1371/journal.pone.0070449 PMC3713062

